# Suppression of joint pain in transient receptor potential vanilloid 4 knockout rats with monoiodoacetate-induced osteoarthritis

**DOI:** 10.1097/PR9.0000000000000951

**Published:** 2021-08-09

**Authors:** Masahiko Soga, Takaya Izumi, Isamu Nanchi, Narumi Horita, Miyuki Yamamoto, Shiori Kawasaki, Koichi Ogawa, Masahide Fujita, Yasuhide Morioka

**Affiliations:** a Department of Pharmacological Efficacy Evaluation, Shionogi TechnoAdvance Research Co. Ltd., Toyonaka, Japan; b Laboratory for Drug Discovery and Disease Research, Shionogi & Co. Ltd., Toyonaka, Japan

**Keywords:** TRPV4, osteoarthritis, monoiodoacetate, dorsal root ganglion

## Abstract

**Introduction:**

Transient receptor potential vanilloid 4 (TRPV4) modulates osteoarthritic (OA) pain in animal models. However, the pathophysiological function of TRPV4 in regulating OA pain remains poorly understood.

**Methods:**

We developed TRPV4-knockout (TRPV4-KO) rats and assessed the effects of *Trpv4* gene deficiency in a monoiodoacetate (MIA)-induced OA pain model (MIA rats) by examining pain-related behavior, pathological changes, and electrophysiological changes in dorsal root ganglion (DRG) neurons. The changes detected in TRPV4-KO rats were confirmed in wild-type rats using a TRPV4 antagonist.

**Results:**

Transient receptor potential vanilloid 4–KO rats showed the same pain threshold as wild-type rats for thermal or pressure stimuli under normal conditions. *Trpv4* gene deletion did not suppress the development of osteoarthritis pathologically in MIA rats. However, the OA-related mechanical pain behaviors observed in MIA rats, including decreased grip strength, increased mechanical allodynia, and reduced weight-bearing on the ipsilateral side, were completely suppressed in TRPV4-KO rats. The DRG neurons in wild-type but not TRPV4-KO MIA rats were depolarized with increased action potentials. Transient receptor potential vanilloid 4 antagonist treatments recapitulated the effects of genetic *Trpv4* deletion.

**Conclusion:**

Transient receptor potential vanilloid 4 was sensitized in the DRG neurons of MIA rats and played a critical role in the development of OA pain. These results suggest that the inhibition of TRPV4 might be a novel potent analgesic strategy for treating OA pain.

## Abstract 2

Supplemental Digital Content is Available in the Text.

Knee joint pain in osteoarthritis model rats is caused by the sensitization of transient receptor potential vanilloid 4 in the dorsal root ganglion neurons

## 1. Introduction

Transient receptor potential vanilloid 4 (TRPV4) is a nonselective cation channel belonging to the TRPV family and is activated by mechanical stimuli,^16,21^ warm temperatures,^27^ hypo-osmotic stress,^15^ and arachidonic acid metabolites, such as 5,6-epoxyeicosatrienoic acid (5,6-EET).^26^ Transient receptor potential vanilloid 4 has been implicated in various types of pain, such as sunburn pain,^17^ temporomandibular arthritic pain,^5^ and neuropathic pain.^1^ In addition, TRPV4 plays an important role in the maintenance of cartilage.^7^ Recently, we found that TRPV4 was sensitized in the knee joints of monoiodoacetate (MIA) rats with increased TRPV4 phosphorylation in the dorsal root ganglion (DRG) neurons and elevated expression of 5,6-EET, the endogenous ligand for TRPV4. Furthermore, the intra-articular administration of a TRPV4 antagonist suppressed pain-related behaviors in MIA rats.^10^ These findings implicated a TRPV4-dependent mechanism in the maintenance of osteoarthritic (OA) pain in the knee joint of MIA rats. However, the pathophysiological function of TRPV4 in the DRG neurons of MIA rats was not examined, and *Trpv4* gene modification was not conducted in our previous study. In this study, we first produced TRPV4-knockout (KO) rats and examined the effects of *Trpv4* gene deficiency in MIA-treated and sham-operated control rats. We further investigated the pathophysiological function of TRPV4 in the DRG neurons of MIA rats electrophysiologically using TRPV4-KO rats and a TRPV4 antagonist.

## 2. Materials and methods

### 2.1. Animals

Male Sprague-Dawley rats (CLEA Japan Inc., Tokyo, Japan) and *Trpv4* gene KO Sprague-Dawley rats (own breeding colony) weighing 210 to 369 g were used. A total of 199 rats (including 88 TRPV4-KO rats) were used. The rats were housed in groups of 3 in plastic cages under controlled temperature and lighting (12-/12-hour light/dark cycle) conditions and provided with food and water ad libitum. All animal behavioral tests were conducted during the light period. The protocols for animal procedures were reviewed and approved by the Animal Care and Use Committee of the Shionogi Pharmaceutical Research Center (Osaka, Japan) and met the Association for Assessment and Accreditation of Laboratory Animal Care International guidelines. The results of the experiments were reported in accordance with the ARRIVE guidelines.^11^

### 2.2. Drugs

The TRPV4 agonists GSK1016790A (Sigma-Aldrich, St. Louis, MO) and 4α-phorbol 12,13-didecanoate (4α-PDD; Sigma-Aldrich) were dissolved in dimethylsulfoxide (DMSO). The TRPV4 antagonist GSK2193874^6^ synthesized in our laboratory was dissolved in 30% DMSO in saline.

### 2.3. Isolation of dorsal root ganglion neurons

Rats were decapitated under deep anesthesia with pentobarbital (60 mg/kg, *i.p.*; Kyoritsu Seiyaku Corporation, Tokyo, Japan). For calcium influx assays, lumbar DRG neurons were collected and incubated in phosphate-buffered saline containing 1 mg/mL collagenase (Sigma-Aldrich) and 1 mg/mL Dispase (Invitrogen, Carlsbad, CA) for 1 hour at 37°C and subsequently treated with 0.05% trypsin-EDTA (Nacalai Tesque, Inc, Kyoto, Japan) for 2 to 3 minutes at 37°C. Alpha-MEM medium (Invitrogen) containing 10% fetal bovine serum (FBS, Invitrogen) was added, and cells were washed twice by centrifugation (1500×*g* for 5 minutes at 20°C). Dorsal root ganglion neurons were suspended in alpha-MEM medium containing 10% FBS, 20 mM HEPES (Gibco, Life Technologies, Carlsbad, CA), a 1% penicillin–streptomycin solution (Nacalai Tesque), 0.5 μg glial cell line–derived neurotrophic factor (Nacalai Tesque), 0.5 μg 2.5S mouse nerve growth factor (Gibco), and 1% supplement N-2 (Invitrogen). For electrophysiological assays, L3 and L4 DRG neurons were isolated and incubated in low-sodium Ringer solution (212.5 mM sucrose, 3 mM KCl, 1 mM NaH_2_PO_4_, 25 mM NaHCO_3_, 11 mM d-glucose, and 5 mM MgCl_2_) containing 2 mg/mL collagenase (Yakult Pharmaceutical Industry Co., Ltd., Tokyo, Japan) for 1 hour at 37°C and subsequently treated with 0.05% trypsin-EDTA for 5 minutes at room temperature. Trituration was gently applied to dissociate neurons from the DRG in culture medium consisting of Dulbecco modified Eagle medium (D6546; Sigma-Aldrich) with 10% FBS, 20 mM HEPES, and a 1% penicillin–streptomycin solution. After centrifugation at 1500×*g* for 5 minutes, DRG neurons were resuspended in culture medium containing 0.5 μg 2.5S mouse nerve growth factor, placed onto glass coverslips precoated with poly-l-lysine and laminin (Gibco), and incubated at 37°C in 5% CO_2_ overnight.

### 2.4. Calcium influx assay of dorsal root ganglion neurons

Dorsal root ganglion neurons cultured in 96-well plates for 7 days were loaded with 5 μM Fluo-4 AM (Dojindo, Kumamoto, Japan) and 0.1% Pluronic F-127 (Molecular Probes, OR) in assay buffer (pH 7.4) consisting of Hanks balanced salt solution (Nissui Pharmaceutical, Tokyo, Japan), 20 mM HEPES (Sigma-Aldrich), and 2.5 mM probenecid for 1 hour at 37°C. Dorsal root ganglion neurons were washed twice with assay buffer and then incubated in assay buffer for 10 minutes at 30°C. An FDSS 7000 functional drug screening system (Hamamatsu Photonics, Shizuoka, Japan) was used to measure calcium influx induced by stimulation with TRPV4 agonists (4α-PDD and GSK1016790 in 0.1% DMSO in assay buffer) and hypotonic solution (218 mOsm) with 480-nm excitation and 540-nm emission wavelengths. The 0.1% DMSO in assay buffer was used as a control for baseline values.

### 2.5. Measurement of pain threshold

The pressure pain threshold was measured using the hind paw withdrawal test with a Randall–Selitto analgesiometer (Ugo Basile, Italy). The mechanical paw withdrawal threshold (g) was determined by the latency time of the hind paw stimulated with the apparatus.^18^ The heat pain threshold was measured using the hot-plate test by placing the rat on the hot plate (Ugo Basile) at 38, 41, and 50°C. The withdrawal latency time (second) was determined by measuring the time for licking, jumping, and escaping from the hot plate.^9^

### 2.6. Monoiodoacetate-induced osteoarthritic pain model

Monoiodoacetate rats were prepared according to our previously described method.^12^ Rats received an intra-articular injection of MIA (Sigma-Aldrich) through the infrapatellar ligament of the right knee joint at a dose of 2 mg in 50 μL of saline. Sham-operated rats received an intra-articular injection of 50 μL of saline into their right knee. The left knee joints remained untreated in all rats. All rats were anesthetized with isoflurane during the procedure.

### 2.7. Measurement of pain-related behaviors in monoiodoacetate rats

Osteoarthritic pain development was assessed by the grip strength test, von Frey hair test, and weight-bearing test. The grip strength test was performed as described previously.^3,12^ The grip strength test was conducted using a grip strength meter (San Diego Instruments, San Diego, CA). In brief, each rat was gently restrained, allowed to grasp the wire mesh frame with its hind limbs, and moved in a rostral-to-caudal direction until the grip released. The mean grip strength was averaged from 2 readings and normalized to body weight. The von Frey hair test (Aesthesio Precision Tactile Sensory Evaluator; Danmic Global, LLC, San Jose, CA) was used as a measure of mechanical hyperalgesia. Rats were placed in a plastic chamber with metal mesh flooring. Ipsilateral hind paw mechanical hyperalgesia was assessed using a modification of the Dixon up-down method.^4^

The difference in weight borne by the ipsilateral compared with the contralateral hind limb was measured using an incapacitance meter (Linton Instrumentation, Norfolk, United Kingdom).^2^ Rats were placed in a plastic chamber designed to have each hind limb resting on a separate transducer pad, which recorded the distribution of the animal's body weight on each limb over 3 seconds. The results were presented as percent body weight on the ipsilateral limb.

### 2.8. Histological analysis of knee joints

The knee joint was isolated from rats under deep anesthesia with 2% to 4% isoflurane, trimmed, and fixed in 4% paraformaldehyde. The joints were decalcified by formic acid and embedded in paraffin. Each joint in a paraffin block was cut at a thickness of 5 µm, and the sections were stained with hematoxylin and eosin or Safranin-O and Fast Green. All procedures for bone decalcification, paraffin embedding, and staining were performed at the Applied Medical Research Laboratory (Osaka, Japan). Cartilage histopathology was assessed using the OARSI cartilage OA histopathology grading system,^2^ and synovitis was assessed using methods described previously.^14^

### 2.9. Electrophysiological examinations

The external recording solution (145 mM NaCl, 2.5 mM KCl, 2 mM BaCl_2_, 1 mM MgCl_2_, 10 mM HEPES, and 11 mM d-glucose) was adjusted to pH 7.4 with NaOH. Patch electrodes were fabricated from borosilicate glass capillaries using an electrode puller (P97; Sutter Instrument Co., Novato, CA). The tip resistances of patch electrodes were 2.4 to 4.6 MΩ when filled with an internal solution (135 mM K-gluconate, 5 mM KCl, 10 mM HEPES, 1.1 mM EGTA, 2 mM MgCl_2_, 3 mM ATP-Mg, and 0.3 mM GTP-Tris adjusted to pH 7.2 with KOH). In the whole-cell configuration, membrane potentials were recorded with an EPC-10 amplifier and PatchMaster software (HEKA, Freiburg, Germany) and digitized at 10 kHz with PowerLab and LabChart software (AD Instruments, Colorado Springs, CO). Dorsal root ganglion neurons sufficiently separated from other culture cells were selected under a microscope. All patch-clamp experiments were performed at room temperature (25°C) or 37°C. The chamber temperature was controlled with a heater controller (TC-344B; Warner Instruments, Hamden, CT) and monitored using a temperature sensor probe. GSK2193874 (1 μM) was bath applied for 1 to 5 minutes.

### 2.10. Statistical analysis

Statistical analyses were performed with GraphPad Prism 9.0 (GraphPad Software, Inc., San Diego, CA). Data are expressed as the mean ± SEM. Comparisons of groups were performed using unpaired *t*-tests, one-way ANOVA, or two-way ANOVA followed by the Tukey test. The Fisher exact test was used in electrophysiological experiments. Values of *P* < 0.05 were considered significant.

## 3. Results

### 3.1. Generation of transient receptor potential vanilloid 4–knockout rats

**Figure 1.**
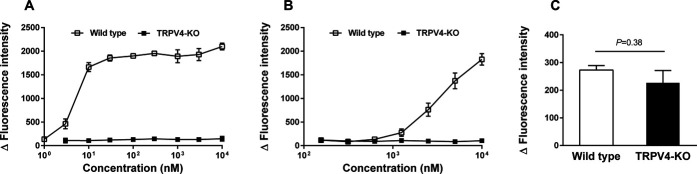
Calcium influx induced by TRPV4 agonists in the DRG neurons of TRPV4-KO and wild-type rats. DRG neurons (1.8×10^5^ neurons/well) were prepared from TRPV4-KO and wild-type rats (n = 3, each). Calcium influx was expressed as the increased fluorescence intensity from the baseline (Δ fluorescence intensity, Ex480/Em540). Each experiment was performed in triplicate. Data are presented as the mean ± SEM. (A) GSK106170, (B) 4α-phorbol 12,13-didecanoate (4α-PDD), and (C) hypotonic solutions (218 mOsm). Data were analyzed by unpaired *t*-tests. DRG, dorsal root ganglion; TRPV4-KO, transient receptor potential vanilloid 4–knockout.

Transient receptor potential vanilloid 4–KO rats were generated using the CRISPR-Cas9 system, and the absence of the gene was confirmed by analyzing DNA sequences (Supplemental Fig. 1, available at http://links.lww.com/PR9/A127). The homozygous KO rats were born according to the Mendelian ratio and showed no noticeable abnormalities. To confirm a functional deficiency in TRPV4, the cellular responses induced by TRPV4 agonists were examined in DRG neurons. Two different TRPV4 agonists, including 4α-PDD and GSK1016790A, induced a dose-dependent increase in calcium influx in DRG neurons from wild-type rats but not in those from TRPV4-KO rats (Figs. 1A, B). These results indicate that the functions of TRPV4 were completely abolished in TRPV4-KO rats. The increase in calcium influx induced by hypotonic stimulation was similar between wild-type and TRPV4-KO rats (Fig. 1C), as previously reported in TRPV4-KO mice.^13^

### 3.2. Effect of transient receptor potential vanilloid 4 deficiency on normal pain thresholds

**Figure 2.**
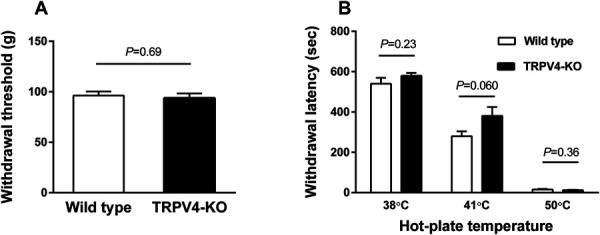
Effects of TRPV4 deficiency on normal pain thresholds. The paw withdrawal thresholds and latencies were measured in TRPV4-KO and wild-type rats. All experiments were performed using 7- to 8-week-old rats. The same rats were used for each test on another day. (A) The mechanical withdrawal thresholds of hind right paws were measured using the Randal–Selitto test. (B) The thermal withdrawal latencies of hind paws were measured with the hot-plate test. The cut-off time was set at 600 seconds. Data are presented as the mean ± SEM (n = 12, each) and were analyzed by unpaired *t*-tests. TRPV4-KO, transient receptor potential vanilloid 4–knockout.

Because TRPV4 is activated by pressure and temperature, we first examined the pressure and thermal pain withdrawal thresholds in TRPV4-KO rats using the hot-plate test and Randall–Selitto test. The pressure and thermal pain withdrawal thresholds were similar between TRPV4-KO rats and wild-type control rats (Randall–Selitto test: *P =* 0.69; hot-plate test: 38°C, *P* = 0.23, 41°C, *P* = 0.060, and 50°C, *P* = 0.36). These results suggest that TRPV4 is not involved in pain sensation to pressure and thermal stimuli under normal conditions (Figs. 2A, B).

### 3.3. Effect of transient receptor potential vanilloid 4 deficiency on pain-related behaviors in monoiodoacetate rats

**Figure 3.**
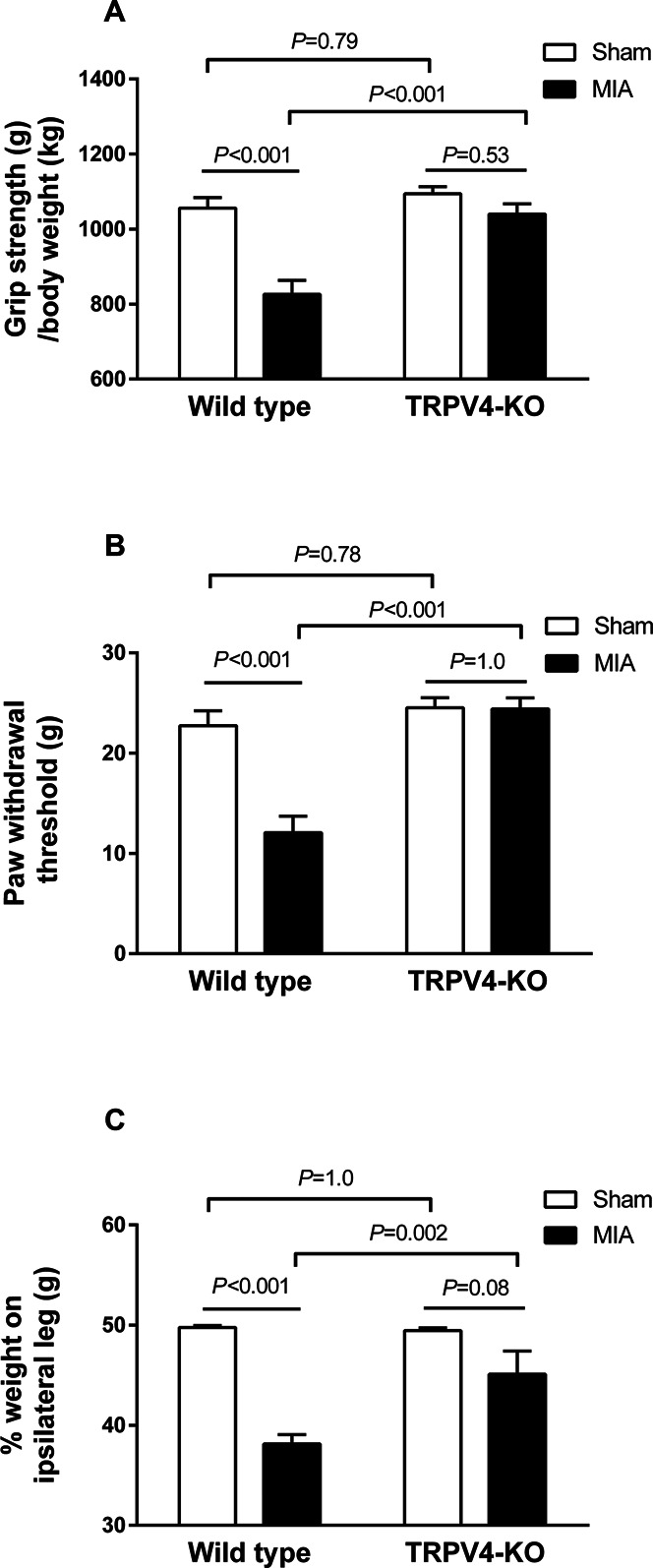
Effects of TRPV4 deficiency on pain-related behaviors in MIA rats. The pain-related behaviors in TRPV4-KO and wild-type rats were assessed on day 14 after saline (sham) or MIA injection. All experiments were performed using 5- to 7-week-old rats at the time of saline (sham) or MIA injection. The same rats were used for the grip strength test and the von Frey filament test on the same day. (A) The grip strengths of the hind limbs were expressed as grip strength (g)/body weight (kg). For wild-type rats, the values were sham: 1056 ± 28, n = 15 and MIA: 826 ± 37, n = 15. For TRPV4-KO rats, the values were sham: 1094 ± 19, n = 15 and MIA: 1040 ± 28, n = 17. The body weights were sham: 306 ± 5 and MIA: 307 ± 6 (*P* = 0.91 vs sham) for wild-type rats and sham: 303 ± 10, *P* = 0.82 vs sham in wild type and MIA: 293 ± 8, *P* = 0.40 vs sham for TRPV4-KO rats. (B) The mechanical allodynia in ipsilateral right hind paws was expressed as the paw withdrawal threshold (g) in the von Frey filament test. For wild-type rats, the values were sham: 23 ± 1, n = 15 and MIA: 12 ± 2, n = 15. For TRPV4-KO rats, the values were sham: 25 ± 1, n = 15 and MIA: 24 ± 1, n = 17. (C) The weight-bearing on ipsilateral right hind limbs was expressed as % weight on the ipsilateral leg. For wild-type rats, the values were sham: 49.8 ± 0.2, n = 10 and MIA: 38.1 ± 0.9, n = 10. For TRPV4-KO rats, the values were sham: 49.4 ± 0.3, n = 9 and MIA: 45.1 ± 2.3, n = 9. Data are presented as the mean ± SEM and were analyzed by a two-way ANOVA followed by the Tukey test. MIA, monoiodoacetate; TRPV4-KO, transient receptor potential vanilloid 4–knockout.

Monoiodoacetate rats were used as an experimental OA pain model, which develops knee OA at 2 weeks after MIA injection and has been previously used to examine the analgesic efficacy of the intra-articular administration of TRPV4 antagonists.^10^ The grip strength test, von Frey test, and static weight-bearing test were used to examine the development of pain and the effects of TRPV4 deficiency. Intra-articular injection of MIA decreased grip strength in wild-type rats compared with that in sham-operated rats at 2 weeks (Fig. 3A). No reduction in grip strength was observed in TRPV4-KO MIA rats (*P* = 0.53). The grip strength threshold was similar between wild-type and TRPV4-KO sham-operated rats (*P* = 0.79) but was significantly different in MIA rats (*P* < 0.001). In addition, the body weights did not differ significantly between sham rats and MIA rats or between wild-type rats and TRPV4-KO rats (Fig. 3A). Mechanical allodynia assessed by the von Frey test was significantly increased in MIA wild-type rats compared with sham-operated rats (12 ± 2 vs 23 ± 1 g, *P* < 0.001). No reduction in the withdrawal threshold was observed in TRPV4-KO MIA rats (*P =* 1.0). The threshold in the von Frey test was similar in wild-type and TRPV4-KO sham-operated rats (*P =* 0.78) but was significantly different in MIA rats (*P* < 0.001, Fig. 3B). The weight-bearing on the ipsilateral hind limb was significantly decreased in MIA wild-type rats compared with sham-operated rats (38.2 ± 0.9% vs 49.8 ± 0.2%, *P* < 0.001) and with TRPV4-KO MIA rats (45.1 ± 2.3%, *P* = 0.002, Fig. 3C). No reduction in weight-bearing on the ipsilateral hind limb was observed in TRPV4-KO MIA rats (*P =* 0.08). The balance in the weight-bearing of each hind limb was normal in TRPV4-KO sham-operated rats (49.4%, Fig. 3C). These findings suggest that TRPV4 plays a critical role in the development of OA pain.

### 3.4. Effect of transient receptor potential vanilloid 4 deficiency on knee joint damage in monoiodoacetate rats

**Figure 4.**
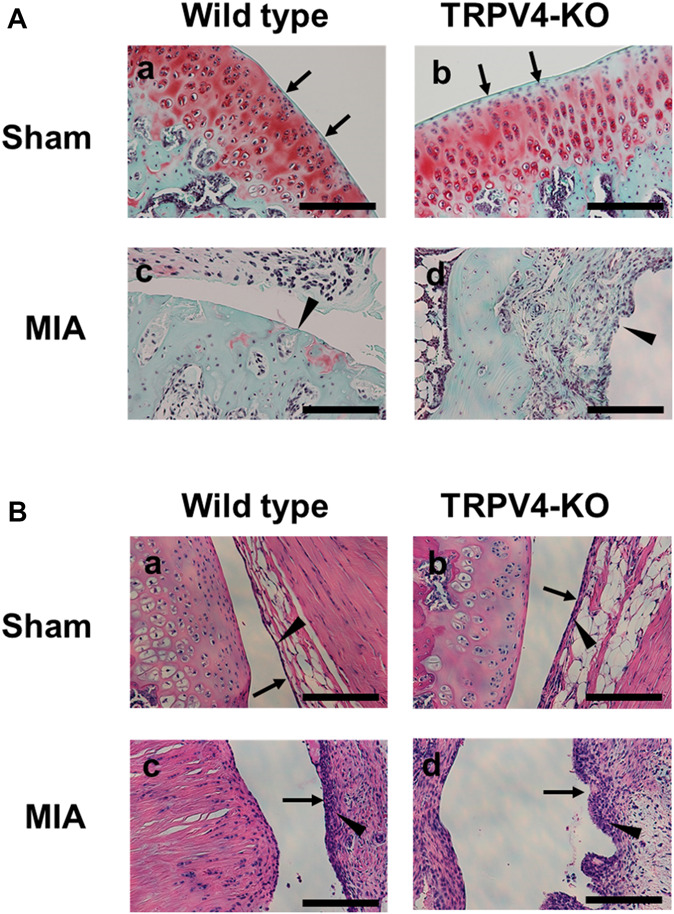
Effects of TRPV4 deficiency on knee joint damage in MIA rats. The knee joint damage in TRPV4-KO and wild-type rats was assessed on day 14 after saline (sham) (a and b) and MIA injection (c and d). Histological analysis was performed by Safranin-O and Fast Green staining for cartilage grading (A) and H&E staining for synovitis scoring (B). (A) The cartilage surface and cartilage erosion, denudation or deformation are indicated by an arrow and a black triangle, respectively. (B) The synovial cell layer and synovial cells are indicated by an arrow and a black triangle, respectively. Representative images are shown. Scale 200 µm. The pain-related behaviors in grip strength tests and knee swelling were shown in Supplementary Fig. 2 (available at http://links.lww.com/PR9/A127) and Table 1, respectively. H&E, hematoxylin and eosin; MIA, monoiodoacetate; TRPV4-KO, transient receptor potential vanilloid 4–knockout.

Given that TRPV4 is expressed in articular cartilage and immune cells,^7^ the effects of TRPV4 deficiency on knee joint damage in MIA-induced OA rats were investigated by histological analysis. Knee joints were prepared from saline (sham)-injected or MIA-injected wild-type and TRPV4-KO rats (Supplementary Fig. 2, available at http://links.lww.com/PR9/A127). Knee joint damages were evaluated by grading articular cartilage and scoring synovitis, which are clinically used as OA progression markers and have been used in MIA rats.^19^ The articular cartilage grade (evaluated by thickening of the articular cartilage) and synovitis score (scored by thickening of the synovial cell layer and density of the cells in the synovial layer) were increased in MIA rats (cartilage grade: *P* < 0.001, synovitis score: *P* < 0.001, Figs. 4A, B and Table 1). Increased articular cartilage grades and synovitis scores were also observed in TRPV4-KO MIA rats (articular cartilage grade: *P* < 0.001, synovitis score: *P* < 0.001, Fig. 4A and B and Table 1). No significant differences were found between wild-type and TRPV4-KO MIA rats (articular cartilage grade: *P* = 0.89, synovitis score: *P* = 0.19, Fig. 4A and B and Table 1). Knee swelling, which is a symptom of knee joint inflammation, was increased in both wild-type and TRPV4-KO MIA rats (increase in the knee diameter of 0.7 ± 0.1 mm in wild-type rats and 0.7 ± 0.2 mm in TRPV4-KO rats, Supplementary Table 2, available at http://links.lww.com/PR9/A127). These results suggest that the reduction in pain-related behavior observed in TRPV4-KO MIA rats is not due to the inhibition of knee damage or inflammation induced by osteoarthritis in MIA rats.

**Table 1:** Histological score of cartilage damage and synovitis in the knee joint of rats.

	Wild type			TRPV4-KO		
	Sham	MIA	*P*	Sham	MIA	*P*
Cartilage grade	0.5 ± 0.2	5.1 ± 0.5	<0.001	1.0 ± 0.5	5.6 ± 0.4	<0.001
Synovitis score	0.0 ± 0.0	3.6 ± 0.6	<0.001	0.1 ± 0.1	4.7 ± 0.4	<0.001

MIA or saline (sham) was injected into the knee joint of rats, and histological analysis of the knee joints was performed 2 weeks after injection. All experiments were performed using 6-week-old rats after MIA or saline (sham) injection. The condition of the articular cartilage surface and cartilage morphology was graded as follows: grade 0 (surface intact and cartilage morphology intact), grade 1 (surface intact), grade 2 (surface discontinuity), grade 3 (vertical fissures), grade 4 (erosion), grade 5 (denudation), and grade 6 (deformation). The condition of synovitis was scored by 2 components: enlargement of the synovial lining cell layer from 0 (thickness 1–2 cells) to 3 (thickness ≥10 cells) and density of the cells from 0 (normal) to 3 (greatly increased). The total score of the 2 components (0–6) was used as an indication of the severity of synovitis. Data are presented as the mean ± SEM (wild-type sham: n = 6 and MIA: n = 8; TRPV4-KO sham: n = 8 and MIA: n = 7). Data were analyzed by a two-way ANOVA followed by the Tukey test in each group. No significant differences were observed between wild-type and TRPV4-KO MIA rats (cartilage grade: *P* = 0.89, synovitis score: *P* = 0.19).

ANOVA, analysis variance, TRPV4-KO, transient receptor potential vanilloid 4-knockout.

### 3.5. Effect of transient receptor potential vanilloid 4 deficiency on neuronal activities in the dorsal root ganglion neurons of monoiodoacetate rats

**Figure 5.**
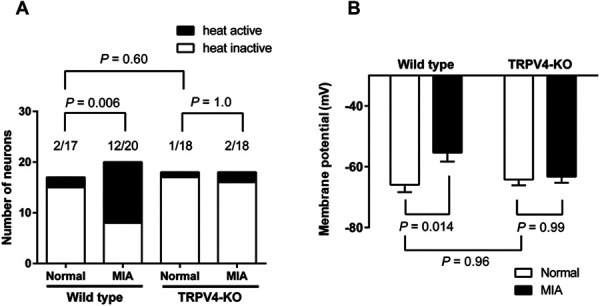
Action potentials in the DRG neurons of TRPV4-KO MIA rats. (A) The number of DRG neurons with an increased (heat active) or unchanged (heat inactive) number of action potentials after warm temperature (37°C) stimulation. Open bars indicate the heat inactive neurons, and black bars indicate heat active neurons. Data were analyzed by the Fisher exact test. The number of action potentials (per 20 seconds) in heat active neurons was normal in wild type: 2.5 ± 1.5, n = 2; MIA in wild type: 16.4 ± 9.1, n = 12; normal in TRPV4-KO: 46, n = 1; and MIA in TRPV4-KO 18 ± 5, n = 2, respectively. Values are the mean ± SEM. The traces of action potentials were shown in Supplementary Fig. 3 (available at http://links.lww.com/PR9/A127). (B) The membrane potential of DRG neurons at 37°C. Open bars indicate the DRG neurons from the control side, and black bars indicate the DRG neurons from the MIA-injected ipsilateral side. Values are the mean ± SEM. For wild types, the values were control: −65.9 ± 2.4 and MIA: −55.4 ± 2.9. For TRPV4-KO rats, the values were control: −64.2 ± 1.8 and MIA: −63.3 ± 2.0. DRG neurons were prepared from 4 rats in one experiment. Data were summarized from 2 to 3 independent experiments in TRPV4-KO rats (n = 8; 18 DRG neurons from the control side and 18 neurons from the MIA-treated side) and wild-type rats (n = 12; 17 DRG neurons from the control side and 20 from the MIA-treated side), respectively. Data were analyzed using a two-way ANOVA followed by the Tukey test. DRG, dorsal root ganglion; MIA, monoiodoacetate; TRPV4-KO, transient receptor potential vanilloid 4–knockout.

Transient receptor potential vanilloid 4 expressed on DRG neurons was sensitized by phosphorylation in MIA rats. We next examined the contribution of DRG neurons to the inhibition of pain in TRPV4-KO rats. Dorsal root ganglion neurons were collected from the ipsilateral side in MIA rats and examined using the patch-clamp system. Dorsal root ganglion neurons from the contralateral side were used as a control. The number of DRG neurons with an increased number of action potentials after warm temperature (37°C) stimulation (thermosensitive DRG neurons) was significantly increased in DRG neurons collected from the ipsilateral side compared with those from the contralateral side of wild-type rats (*P* = 0.006, normal: 12%, 2/17 neurons; MIA: 60%, 12/20 neurons, Fig. 5A). This increase in thermosensitive DRG neurons was not observed in TRPV4-KO MIA rats (*P* = 1.0, Fig. 5A). The membrane potential of DRG neurons from the ipsilateral side of wild-type rats was significantly depolarized compared with that in DRG neurons from the contralateral side of wild-type rats at 37°C (*P* = 0.014, Fig. 5B). No difference in the depolarization of DRG neurons was observed in the ipsilateral and the contralateral side of TRPV4-KO rats (*P* = 0.99, Fig. 5B). The number of thermosensitive neurons and the membrane potential in DRG neurons from the contralateral side were not significantly different between wild-type rats and TRPV4-KO rats (*P* = 0.60 and *P* = 0.96, respectively). These results suggest that the DRG neurons in MIA rats were sensitized and depolarized at body temperature through a TRPV4-dependent mechanism.

### 3.6. Effect of transient receptor potential vanilloid 4 antagonism on pain-related behaviors in monoiodoacetate rats

**Figure 6.**
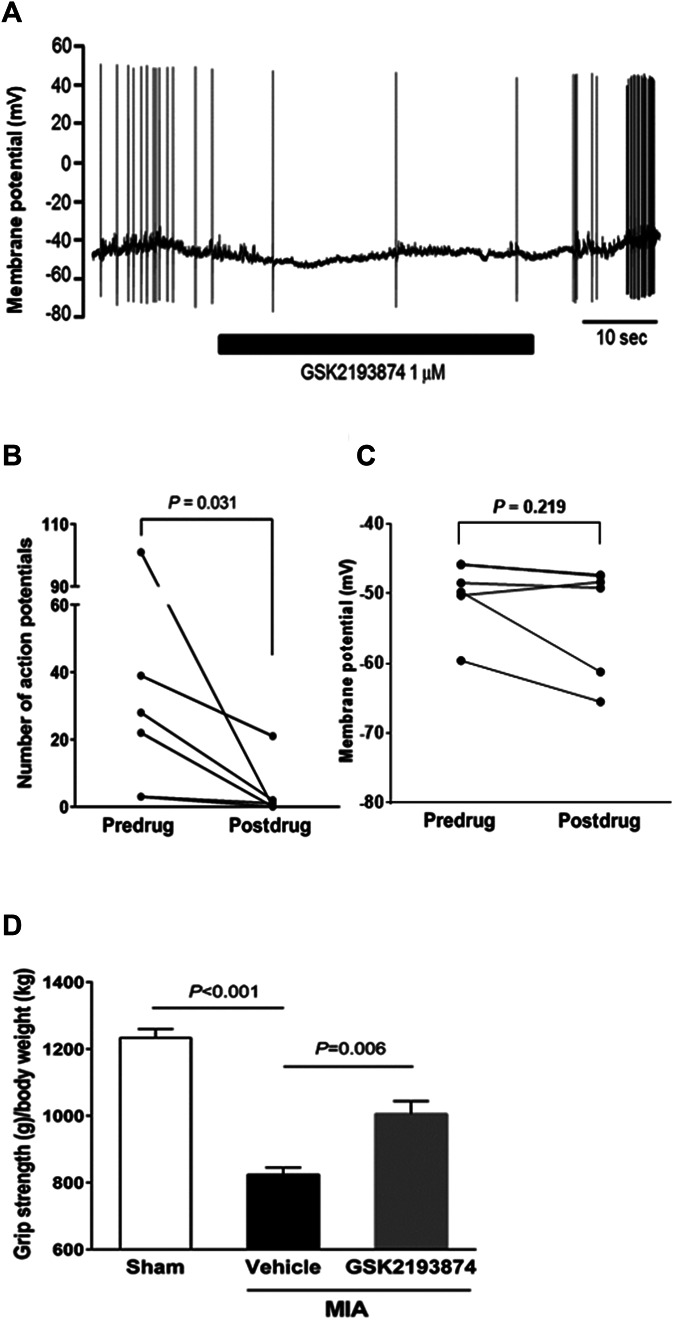
Inhibitory effects of TRPV4 antagonism on increased action potentials in the DRG neurons of MIA rats and pain-related behavior in MIA rats. (A) Representative traces of action potentials in the DRG neurons of MIA rats. Bold line indicates the period of TRPV4 antagonist GSK2193874 treatment. (B) The number of action potentials recorded in DRG neurons in MIA rats before (predrug: 32.7 ± 13.5) and during (postdrug: 4.0 ± 3.1) TRPV4 antagonist treatment. The number of action potentials was counted for 20 seconds at 37°C. (C) The membrane potentials of DRG neurons in MIA rats before (predrug: −50.0 ± 1.9) and during (postdrug: 53.2 ± 3.0) TRPV4 antagonist treatment. DRG neurons were prepared from 4 rats and pooled for experiments. In total, 6 DRG neurons were used. Data were analyzed using the Wilcoxon matched-pairs signed-rank test. (D) The grip strengths of the hind limbs in sham and MIA rats were measured on day 14 after saline (sham) or MIA injection. The experiments were performed using 5- to 7-week-old rats injected with saline (sham) or MIA injection. The grip strengths are expressed as grip strength (g)/body weight (kg). The selection criterion for MIA rats was <950 grip strength (g)/body weight (kg). GSK2193874 or the vehicle control was orally administered to MIA rats, and grip strengths were measured at 3 hours after treatment. The values were sham: 1233 ± 27, n = 6; vehicle: 824 ± 21, n = 4; and GSK2193874: 1005 ± 38, n = 6. Data were analyzed by a one-way ANOVA followed by the Tukey test and are presented as the mean ± SEM. DRG, dorsal root ganglion; MIA, monoiodoacetate; TRPV4, transient receptor potential vanilloid 4.

Given that TRPV4 deficiency would affect the function of signaling molecules downstream of TRPV4, we next examined the effects of the TRPV4-selective antagonist GSK2193874 to clarify the involvement of the ion channel function of TRPV4 in regulating the changes observed in DRG neurons. The action potentials in thermosensitive DRG neurons were immediately inhibited by GSK2193874, and the number of action potentials in MIA rats was significantly suppressed by GSK2193874 (*P* = 0.031, Figs. 6A, B) without affecting membrane potentials (Fig. 6C). These results suggest that the ion channel function of TRPV4 is directly involved in the sensitization of DRG neurons in MIA rats. To confirm the direct involvement of TRPV4 channel function in regulating OA pain, we examined the analgesic effect of GSK2193874 by systematic administration. Oral administration of GSK2193874 immediately and significantly suppressed the pain-related behavior assessed by the grip strength test (*P* = 0.006, Fig. 6D). This result suggests that TRPV4 channel function is directly involved in the regulation of OA pain.

## 4. Discussion

In this study, we first produced TRPV4-KO rats and evaluated the involvement of TRPV4 in knee OA pain using MIA rats. We found that *Trpv4* gene deletion inhibited the development of OA pain in MIA rats and the number of action potentials in the DRG neurons of MIA rats. We also demonstrated that a TRPV4 antagonist recapitulated the effects of *Trpv4* gene deletion. These findings suggest a distinct mechanism and important contribution of TRPV4 to knee OA pain.

We recently reported that 5,6-EET, which is an endogenous agonist for TRPV4, was increased in the knee joints of MIA rats. Furthermore, TRPV4 was sensitized by phosphorylation in the DRG of MIA rats, and intra-articular injection of TRPV4 antagonists suppressed knee pain in MIA rats.^10^ However, the pathophysiological and functional changes of TRPV4 in the DRG neurons of MIA rats remained unknown. To address this, we investigated the role of TRPV4 using the novel TRPV4-KO rat model that we developed.

To the best of our knowledge, this is the first study to examine the behavioral features of TRPV4-KO rats. TRPV4 is activated by mechanical stimuli and body temperatures in vitro. However, under normal conditions, the pain withdrawal thresholds of TRPV4-KO mice and wild-type controls in the hot-plate test (noxious heat) and von Frey test (touch sensation) reported in previous studies^21,25^ were similar to those in our study (Supplementary Fig. 4, available at http://links.lww.com/PR9/A127). In this study, we examined the mechanical pain withdrawal threshold using not only the von Frey test but also the Randall–Selitto test^18^ and grip strength test,^12^ which provide a higher intensity of mechanical pressure stimulation on paw and knee joints, respectively. We confirmed that under normal conditions, the pain thresholds to the hot plate and pressure were normal in TRPV4-KO rats, suggesting that TRPV4 may not affect the warning system for pain under these conditions. Interestingly, the calcium influx in DRG neurons prepared from lumbar DRG neurons induced by hypotonic stimulation was similar between wild-type and TRPV4-KO rats. A previous study showed that the TRPV4 osmosensitivity was low in lumbar DRG neurons.^13^ In this study, we examined only the sensitivity in hind knees or paws innervated by lumbar DRG neurons. Therefore, further studies are required to examine the function of TRPV4 in other regions of the body under normal conditions.

To evaluate OA-related pain, we used the grip strength test (assesses movement-induced knee joint pain), static weight-bearing test (assesses knee joint pain while standing), and von Frey test (assesses mechanical allodynia). These behavioral tests assess mechanical-induced pain and are the most common methods for evaluating OA-related pain in MIA rats to predict clinical OA pain.^20^ Mechanical allodynia is caused by injury to the DRG in MIA rats.^24^ The interactions between the sensory ending of nerves and the DRG with TRPV4 are known to be involved in neuropathic pain.^1^ The mechanical allodynia in TRPV4-KO MIA rats would also be suppressed by the inhibition of TRPV4-dependent DRG activation. As a limitation of our behavioral studies, we did not examine the effects of TRPV4 deficiency on thermal hypersensitivity because MIA rats did not develop thermal hypersensitivity in our previous study,^19^ and it is also not common in OA-related pain in MIA rats.^20^ In addition, we did not assess other OA pain models, such as meniscal transection–induced OA, because the pain-related behaviors in this model were limited in our previous study.^9^ Further studies are required to confirm the involvement of TRPV4 in OA pain using other behavioral tests and animal models.

In this study, we first demonstrated that OA-related mechanical pain did not develop in TRPV4-KO MIA rats, although the extent of synovial inflammation and cartilage destruction was the same as in wild-type control rats. A previous study that investigated temporomandibular joint pain using TRPV4-KO mice also demonstrated that the pain behavior induced by temporomandibular joint inflammation was suppressed in TRPV4-KO mice, but the degree of bone loss and synovial inflammation was similar between KO mice and wild-type controls.^5^ This suggests that the function of TRPV4 in regulating inflammation and cartilage destruction is quite limited in knee OA, although TRPV4 is expressed in inflammatory cells and chondrocytes.

Dorsal root ganglion neurons are responsible for transmitting pain signals from the knee joints to the brain, and TRPV4 expressed on DRG neurons was sensitized by phosphorylation in MIA rats.^10^ Therefore, we next examined the functional changes of DRG neurons in TRPV4-KO MIA rats. We first demonstrated that the DRG neurons from the ipsilateral MIA-treated side were depolarized, and the frequency of action potentials was significantly increased compared with that in the DRG neurons from the contralateral control side. These electrophysiological changes were not observed in TRPV4-KO rats. The membrane potential and spontaneous activity in contralateral control DRG neurons were similar between TRPV4-KO rats and wild-type control rats. These electrophysiological characteristics observed in DRG neurons were consistent with the pain-related behavior observed in TRPV4-KO rats. This finding was also consistent with our previous study, which showed increased TRPV4 phosphorylation in the DRG neurons of MIA rats. Furthermore, we demonstrated that a TRPV4 antagonist could suppress the increased action potential frequency in the DRG neurons of MIA rats. This finding suggests that the increased action potential frequency and depolarization in the DRG neurons of MIA rats are induced directly through TRPV4 and not through the signaling of downstream molecules affected by TRPV4 deficiency. Recently, it was reported that the depolarization induced by the TRPV1 agonist capsaicin was mediated by the interaction between TRPV1 and the Ca-dependent Cl channel anoctamin 1, which caused anion efflux–mediated depolarization in DRG neurons.^22^ Anoctamin 1 can also interact with TRPV4 functionally and temperature dependently in salivary gland acinar cells^8^ and is co-expressed with TRPV4 in DRG neurons.^23^ The TRPV4-dependent increase in action potential frequency and depolarization in the DRG neurons of MIA rats might be mediated by TRPV4–ANO1 interactions. Because TRPV4 is activated at physiological body temperature, the spontaneous action potentials would be increased in the DRG neurons of MIA rats in which TRPV4 was sensitized by phosphorylation. TRPV1 was also sensitized in the DRG neurons of MIA rats.^12^ However, TRPV1 is opened at higher temperatures compared with TRPV4. Therefore, it is likely that TRPV4 is mainly involved in the increased action potential frequency in the DRG neurons of MIA rats. Further study is required to directly compare the involvement of TRPV1 and TRPV4 in OA knee pain.

In this study, we first demonstrated that the systematic administration of a TRPV4 antagonist inhibited OA-induced pain. Transient receptor potential vanilloid 4 antagonist GSK2193874 significantly inhibited the increased action potentials in the DRG neurons of MIA rats. However, its analgesic effects in MIA rats were less potent compared with those observed in TRPV4-KO rats. The multimodal antagonist effects of this compound were not reported. The tissue concentration or the multimodal antagonist effects on TRPV4 may be insufficient. Although membrane potential and action potential shape were not changed by GSK2193874 (Supplementary Fig. 5, available at http://links.lww.com/PR9/A127), as a limitation, we cannot exclude the possibility of off-target effects of GSK2193874. Further study is required to examine the effects of TRPV4 antagonists as a new medicine for the treatment of OA pain.

In conclusion, the DRG neurons of MIA rats were depolarized with increased action potentials mediated by TRPV4. Complete genetic inhibition of *Trpv4* evidently suppressed OA pain without affecting normal pain thresholds. Transient receptor potential vanilloid 4 antagonism mimicked the effects of genetic *Trpv4* deletion. These findings enhance our understanding of TRPV4 function in regulating OA pain and the use of TRPV4 antagonists as novel potent analgesics for OA pain.

## Disclosures

All funding was provided by Shionogi & Co., Ltd. M. Soga and N. Horita were employees of Shionogi TechnoAdvance Research Co. Ltd. T. Izumi, I. Nanchi, M. Yamamoto, S. Kawasaki, K. Ogawa, M. Fujita, and Y. Morioka were employees of Shionogi & Co., Ltd. at the time of data collection and manuscript writing.

## Appendix A. Supplemental digital content

Supplemental digital content associated with this article can be found online at http://links.lww.com/PR9/A127.

## Supplementary Material

SUPPLEMENTARY MATERIAL
