# Supplementary material for: Suppression of joint pain in transient receptor potential vanilloid 4 knockout rats with monoiodoacetate-induced osteoarthritis
Source: Pain Rep. 2021 Aug 9;6(3):e951. doi: 10.1097/PR9.0000000000000951 (PMC8357256; doi:10.1097/PR9.0000000000000951)
Supplement: SUPPLEMENTARY MATERIAL [file painreports-6-e951-s001.pdf]

Supplementary Information

Supplementary Methods

Generation of TRPV4-KO rats

The TRPV4-KO rat model was generated using a pronuclear injection procedure. A mixture of spCas9 mRNA (50 ng/µl) and short guide RNA (10 ng/µl) was injected into the pronucleus of fertilized eggs derived from Sprague–Dawley rats. The KO founder rat with a 7-bp deletion in exon 9 of the *Trpv4* gene was identified by PCR and sequencing analysis using genomic DNA from the ear punch. Homozygous KO rats were generated through the inbreeding of heterozygous rats. Rats had no abnormalities in growth or reproductive function. The nucleic acid sequences used for the preparation of *Trpv4*-KO rats were as follows: target sequence of short guide RNA, 5'-accctacccttaccgtaccacgg-3'; PCR forward primer, 5'-tgctgaggacacagatgtttgg-3'; and PCR reverse primer, 5'- agacggtgagaggaatatggagc-3' (Supplementary Fig. 1).

A

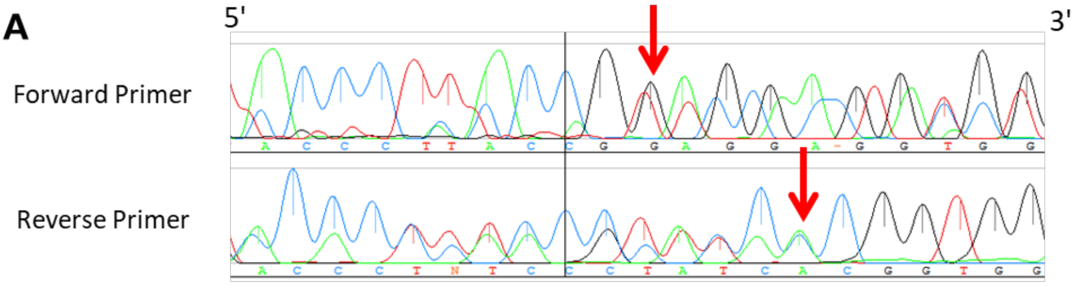

B

rat Trpv4 Exon9

wildtype allele CCACCCTACCCTTACCGTACCACGGTGGACTACCTGAGGCTGGCTGGT

knockout allele CCACCCTACCCTTAC-------CGGTGGACTACCTGAGGCTGGCTGGT

Supplementary Figure 1. Sequence of the *Trpv4*-KO allele. (A) *Trpv4*-KO allele was confirmed by genomic sequencing in KO founder rats. The double peaks indicated with arrows show that the founder was heterozygous with one wild-type allele and one KO allele. Primer sequences were as follows: forward, 5'-tgctgaggacacagatgtttgg-3', and reverse, 5'-agaagccagactggagtaggag-3'. (B) Sequence analysis showed that the KO allele had a 7-bp deletion in exon 9, indicating that the TRPV4 protein was deleted by a frameshift mutation.

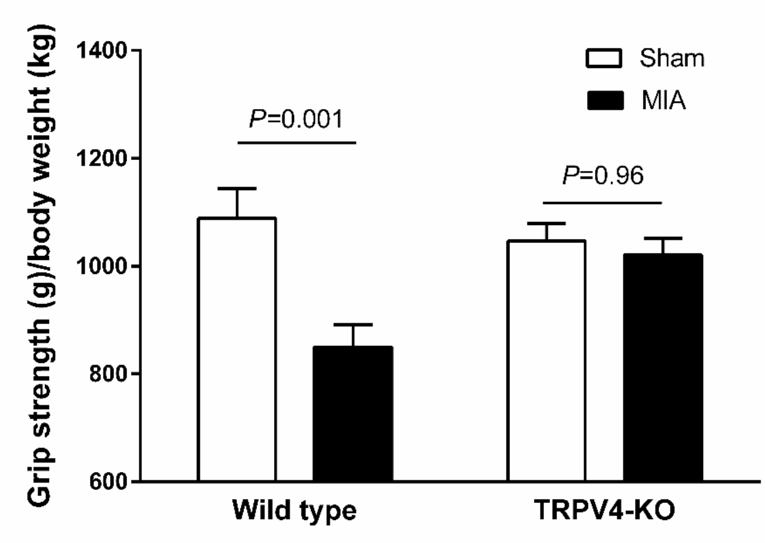

Supplementary Figure 2. Effects of TRPV4 deficiency on pain-related behaviors in MIA rats. The pain-related behaviors in TRPV4-KO and wild type rats were assessed at 2 weeks after saline (sham) or MIA injection. All experiments were performed using 6-week-old rats at MIA or saline (sham) injection. The grip strengths of the hind limbs are expressed as grip strength (g)/body weight (kg). On day 14, for wild types, the values were sham: 1088 ± 55, n=7 and MIA: 850 ± 41, n=10. For TRPV4-KO rats, the values were sham: 1047 ± 32, n=10 and MIA: 1021 ± 31, n=10. Data are presented as the mean ± SEM and were analyzed by a two-way ANOVA followed by Tukey's test.

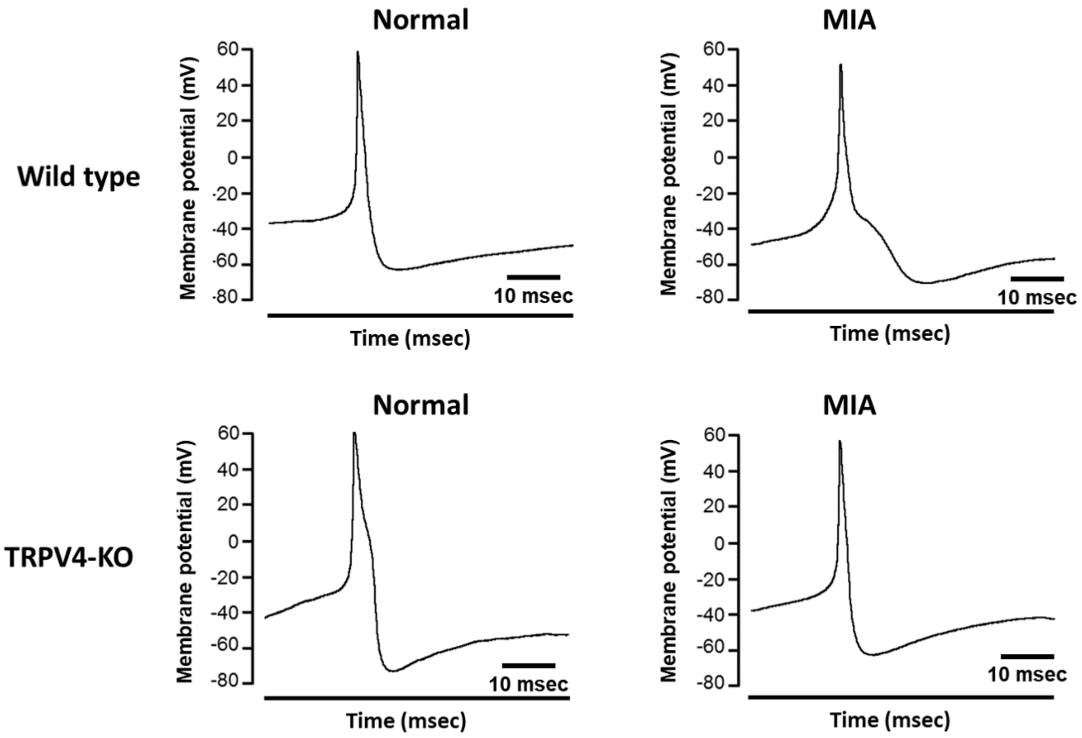

Supplementary Figure 3. Representative shapes of the action potentials of heat active DRG neurons shown in Figure 5.

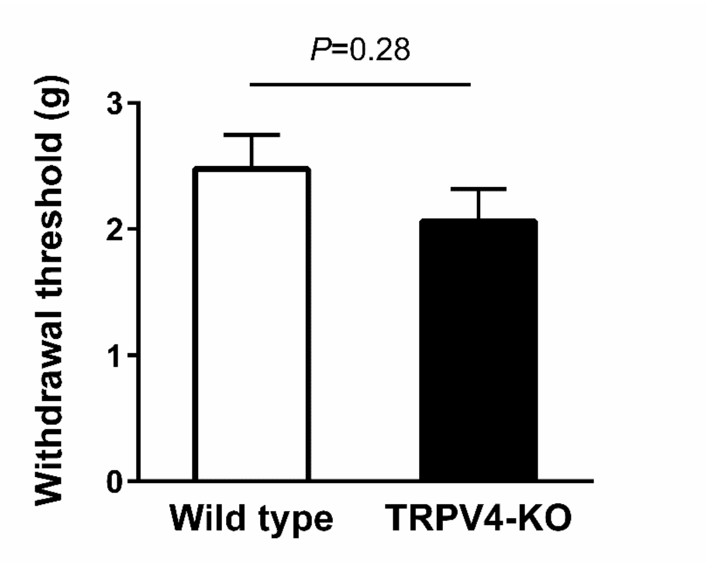

Supplementary Figure 4. Normal touch sensation in TRPV4-KO mice. Male C57BL/6J TRPV4-KO mice that were 8–9 weeks of age and age-matched wild type male mice were obtained from our breeding colony [1]. The right hind paw withdrawal threshold was measured using the von Frey hair test. Data are presented as the mean ± SEM (n=16, each) and were analyzed by unpaired *t*-tests.

Supplementary reference

1. Kawasaki, M. Soga, I. Nanchi, M. Yamamoto, S. Imai, T. Takahashi, N. Tsuno, T. Asaki, Y. Morioka, M. Fujita, S. (accepted on European Journal of Pharmacology)

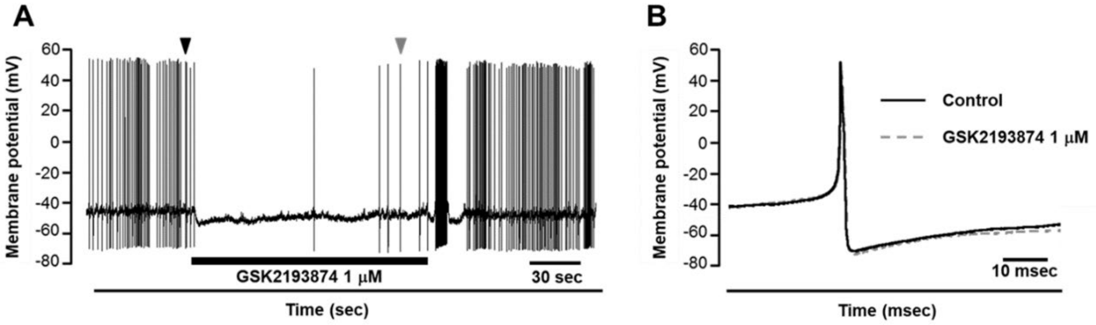

Supplementary Figure 5. Effects of GSK2193874 on the shape of action potentials of heat active DRG neurons. DRG neurons were prepared from four MIA-injected ipsilateral side of MIA rats in wild type and pooled for experiments. In total, eight rats were used. (A) Representative trace of action potentials of heat active DRG neuron. Bold line indicates the period of TRPV4 antagonist GSK2193874 treatment. Black and grey triangle indicate the action potential used in Supplementary Figure 5B as before and during GSK2193874 treatment, respectively. (B) The shape of action potentials before (black line) and during (grey dashed line) GSK2193874 treatment.

	Wild type		TRPV4-KO	
	Sham	MIA	Sham	MIA
Body weight (g)	305 ± 4	299 ± 7	337 ± 8	318 ± 9
Knee diameter (mm)	6.6 ± 0.1	7.3 ± 0.1<br>(Δ 0.7± 0.1)	7.3 ± 0.3	8.0 ± 0.2<br>(Δ 0.7± 0.2)

Supplementary Table 1. Effects of TRPV4 deficiency on knee swelling in MIA rats. The knee diameter of the ipsilateral leg was determined using a digital caliper (CD-15CX; Mitutoyo Corporation, Kanagawa, Japan) at 2 weeks after saline (sham) and MIA injection. Data are presented as the mean ± SEM. The increase in knee diameter in MIA rats was determined by subtracting the sham knee diameter from the MIA knee diameter at each time point in each group and presented in parentheses.
